# Dopamine receptor antagonist thioridazine inhibits tumor growth in a murine breast cancer model

**DOI:** 10.3892/mmr.2015.3967

**Published:** 2015-06-22

**Authors:** TAO YIN, SISI HE, GUOBO SHEN, TINGHONG YE, FUCHUN GUO, YONGSHENG WANG

**Affiliations:** 1 State Key Laboratory of Biotherapy/Collaborative Innovation Center of Biotherapy, West China Hospital, Chengdu, Sichuan 610041, P.R. China; 2 Department of Thoracic Oncology, Cancer Center, West China Hospital, Sichuan University, Chengdu, Sichuan 610041, P.R. China

**Keywords:** thioridazine, breast cancer, angiogenesis

## Abstract

Neuropsychological factors have been shown to influence tumor progression and therapeutic response. The present study investigated the effect of the dopamine receptor antagonist thioridazine on murine breast cancer. The anti-tumor efficacy of thioridazine was assessed using a murine breast cancer model. Cell apoptosis and proliferation were analyzed *in vitro* using flow cytometry (FCM) and the MTT assay, respectively. Western blot analysis was performed to assess Akt, phosphorylated (p)-Akt, signal transducer and activator of transcription (STAT) 3, p-STAT3 and p-p65 in tumor cells following treatment with thioridazine. The Ki67 index and the number of terminal deoxynucleotidyl transferase dUTP nick end labeling (TUNEL)-positive apoptotic cells were assessed in the tumor sections. Thioridazine was found to reduce tumor growth, inhibit tumor cell proliferation and induce apoptosis in a dose- and time-dependent manner *in vitro*. Thioridazine was also found to markedly inhibit tumor proliferation and induce tumor cell apoptosis *in vivo* as shown by the lower Ki67 index and increase in TUNEL-positive cells. In addition, thioridazine was observed to inhibit the activation of the canonical nuclear factor κ-light-chain-enhancer of activated B cells pathway and exert anti-tumor effects by remodeling the tumor stroma, as well as inhibit angiogenesis in the tumor microenvironment. In conclusion, thioridazine was found to significantly inhibit breast tumor growth and the potential for thioridazine to be used in cancer therapy may be re-evaluated and investigated in clinical settings.

## Introduction

Neuropsychological stimulation has been shown to affect tumor progression and therapeutic response. Neural stimulation is associated with increased tumor incidence and metastasis (1). β-adrenergic activation of the cyclic adenosine monophosphate-protein kinase A signaling pathway has been reported to enhance tumor angiogenesis *in vivo* and promote malignant cell growth (2). β-blockers have been shown to reduce recurrence rates and mortality in patients with breast, melanoma and prostate cancer (3–5). A recent report found that cholinergic parasympathetic signaling regulates prostate cancer invasion (6). Furthermore, transplantation of beta-endorphin neurons into the hypothalamus has been reported to increase the activity of natural killer (NK) cells and macrophages, reduce inflammation and epithelial to mesenchymal transition (EMT) in tumor tissues and suppress mammary tumor growth and progression (7). Neuropsychological factors also influence the effect of anticancer therapies. Nonmyelinating Schwann cells are components of a hematopoietic niche and maintain hematopoietic stem cell (HSC) hibernation (8). Sympathetic nerves in the marrow of mice promote the survival of constituents of the stem cell niche which initiate recovery, thus chemotherapy-induced nerve injury in the bone marrow has been found to impair hematopoietic regeneration (9). Adrenergic nerve protection strategies, for example the administration of 4-methylcatechol or glial-derived neurotrophic factor, have been reported to promote hematopoietic recovery (9).

Dopamine receptors (DRs) are involved in several physiological and pathological processes, including age, emotion, opiate addiction and vascular activity. Five DRs have been identified and divided into two families: The D1 (including D1R and D5R) and D2 (including D2R, D3R and D4R) receptor families (10). DRs expressed on lymphocytes are involved in pathogenesis. D3R expressed on CD4^+^ T cells has also been reported to have an important role in the pathogenesis of 1-methyl-4-phenyl-1,2,3,6-tetrahydropyridine-induced Parkinson's disease (11). D3R-deficient mice were observed to be protected against the loss of dopaminergic neurons and microglial activation during MPTP-induced Parkinson's disease; however, upon transfer of wild-type CD4^+^ T cells, mice became susceptible to MPTP-induced neuronal degeneration and activation of microglia (11). The role of dopamine, not only in mediating interactions in the nervous system, but as an immunomodulator in the pathogenesis of disease, has been the focus of much research (12). Circulating dopamine levels have been reported to be higher in patients with lung cancer compared with healthy donors and dopamine has been found to effectively inhibit proliferation and cytotoxicity in T lymphocytes through D1 DRs (13).

Sachlos *et al* (14) identified that DRs are expressed in lymphoid stem cells and CD44^+^ CD24^−^/low breast cancer stem cells (CSCs). However, DRs have not been observed in primitive HSCs or progenitor populations. Differential DR expression enables potential DR drug targeting for cancer (14). Thioridazine is a well established anti-psychotic and -anxiety agent which acts through DR1-5 (15). The present study aimed to investigate the anti-tumor effect of thioridazine in a murine breast cancer model.

## Materials and methods

### Cell culture

The 4T1 cancer cell line (American Type Culture Collection, Manassas, VA, USA) was maintained in RPMI-1640 (Gibco-BRL, Carlsbad, CA, USA) supplemented with 10% fetal bovine serum at 37°C in a humidified atmosphere containing 5% CO_2_.

### Cell cycle and apoptosis analysis

The 4T1 cell line was treated with 5, 10 and 20 *µ*M thioridazine. To assess apoptosis, cells were collected and stained with fluorescein isothiocyanate (FITC)-labeled Annexin V and propidium iodide, according to the manufacturer's instructions (Roche, Mannheim, Germany). Apoptotic cells were analyzed using flow cytometry (FCM) using CellQuest™ software (BD Biosciences, San Jose, CA, USA). Thioridazine was purchased from Sigma-Aldrich (St. Louis, MO, USA).

### MTT assay

MTT assay was performed in 96-well plates to determine cell growth inhibition. A total of 3,000 cells/well were seeded in 96-well plates and treated with 20 *µ*M thioridazine the following day. After 24–72 h drug incubation, MTT was added to each well and incubated for 4 h at 37°C. The supernatant was then removed and formazan precipitates in the cells were dissolved using 150 *µ*l dimethyl sulfoxide. Absorbance was read at 570 nm and a growth curve was plotted.

### Western blot analysis

Subsequent to treatment with 20 *µ*M thioridazine for 24 h, 4T1 tumor cells were harvested and lysed using radioimmunoprecipitation assay buffer containing protease inhibitors. Equal quantities of protein were loaded and resolved using SDS-PAGE and transferred onto polyvinylidene difluoride membranes. Membranes were then incubated with primary antibodies against STAT3, phosphorylated (p)-STAT3, Akt, p-Akt and p-p65 (all rabbit mAb; cat. nos. 4904, 9145, 4685, 4051 and 3033 respectively; Cell Signaling Technology Inc., Danvers, MA USA), followed by incubation with horseradish peroxidase-conjugated secondary antibodies (Goat pAb; cat. no. SP-9001; ZSGB-BIO ORIGENE, Beijing, China). Immunoreactive bands were visualized using an enhanced chemiluminescence detection system.

### Tumor challenge and treatment

In order to determine the anti-tumor activity of thioridazine, flank syngeneic breast tumors were established in six- to eight-week-old female BALB/c mice through subcutaneous injection with 1×10^6^ 4T1 cells. When the tumors reached ~100 mm^3^ on day six following inoculation, the mice with established tumors were stratified by tumor volume and randomly divided into two groups, which were treated with vehicle and thioridazine (32 mg/kg body weight), respectively. Thioridazine was intraperitoneally administered daily. Tumor diameters were measured using a caliper and tumor volumes were calculated by the formula 0.52 × a ×b^2^, where a is the larger diameter and b is the smaller diameter.

### Immunohistochemistry

Tumors were harvested and paraffin-embedded, then tumor sections were stained with hematoxylin and eosin. For the analysis of cell proliferation, tumor sections were incubated with anti-Ki67 antibodies (cat. no. AB9260; Millipore Corporation, Billerica, MA, USA) at 4°C overnight, followed by incubation with biotin-conjugated secondary antibodies and streptavidin-horseradish peroxidase complexes. For the analysis of apoptosis, the TUNEL assay (Promega Corporation, Madison, WI, USA) was performed on the tumor sections, according to the manufacturer's instructions. Frozen tumor sections were incubated with CD31 antibodies (cat. no. ab7388; Abcam PLC, Cambridge, UK) and phycoerythrin-conjugated secondary antibodies (cat. no. ab7010; Abcam PLC) for microvessel analysis.

### Statistical analysis

Data are presented as the mean ± standard deviation. Statistical comparisons were performed using analysis of variance or the student's t-test. SPSS V13.0 software (SPSS Inc., Chicago, IL, USA) was used for statistical analysis. P<0.05 was considered to indicate a statistically significant difference.

## Results

### Dopamine receptor antagonist thioridazine reduces breast tumor growth

**Figure 1:**
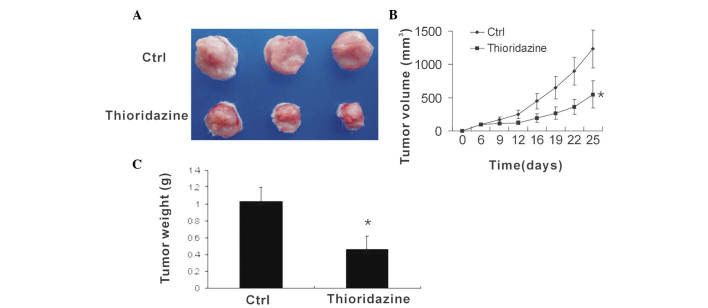
Dopamine receptor antagonist thioridazine reduces breast tumor growth. (A) Tumors from each group at day 25 post-inoculation. (B) Tumors were measured regularly and the relative tumor volumes were calculated. (C) Tumor weight was measured at day 25 post-inoculation and thioridazine significantly reduced tumor volume and weight. ^*^P<0.05 vs. ctrl. Ctrl, control.

To determine the anti-tumor efficacy of thioridazine *in vivo*, flank syngeneic 4T1 breast tumors were established in BALB/c mice. The mice with then treated with vehicle or thioridazine. The control tumors were observed to grow rapidly, with a volume of 1232.8±282.8 mm^3^ at day 25 post-inoculation. However, following treatment with thioridazine, the tumor volume was found to be 549.3±205.2 mm^3^ (Fig. 1A and B). Thus, thioridazine reduced tumor volume by 55% (Fig. 1C), suggesting that the dopamine receptor antagonist thioridazine effectively reduces breast tumor growth.

### Thioridazine induces apoptosis and inhibits tumor cell growth in vitro

**Figure 2:**
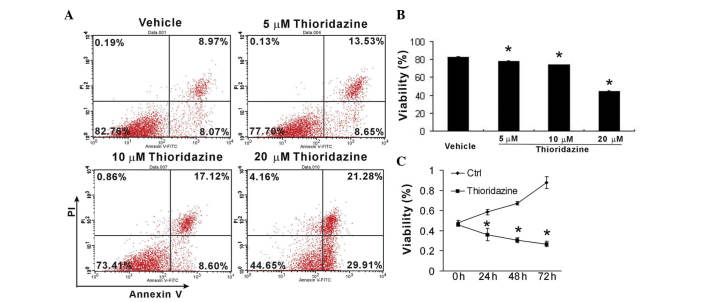
Thioridazine induces tumor cell apoptosis and inhibits tumor cell proliferation. (A and B) 4T1 tumor cells were treated for 24 h with 5, 10 and 20 *µ*M thioridazine. Tumor cell apoptosis was assessed using flow cytometry. Thioridazine induced cell apoptosis in a dose-dependent manner. (C) 4T1 tumor cells were treated with 20 *µ*M thioridazine for 24, 48 and 72 h. Tumor cell viability was determined using the MTT assay. Thioridazine inhibited tumor cell proliferation in a time-dependent manner. ^*^P<0.01 vs. ctrl. Ctrl, control.

To investigate the cytotoxic and cytostatic effects of thioridazine on breast cancer cells, murine 4T1 cells were treated with various concentrations of thioridazine for 24 h. Cell apoptosis was then analyzed using FCM. Thioridazine was observed to markedly induce 4T1 cell apoptosis in a dose-dependent manner (Fig. 2A and B). Furthermore, an MTT assay revealed that thioridazine significantly inhibited tumor growth (Fig. 2C). These findings suggested that thioridazine effectively reduced tumor cell growth and induced tumor apoptosis.

### Thioridazine induces apoptosis and inhibits tumor cell growth in vivo

**Figure 3:**
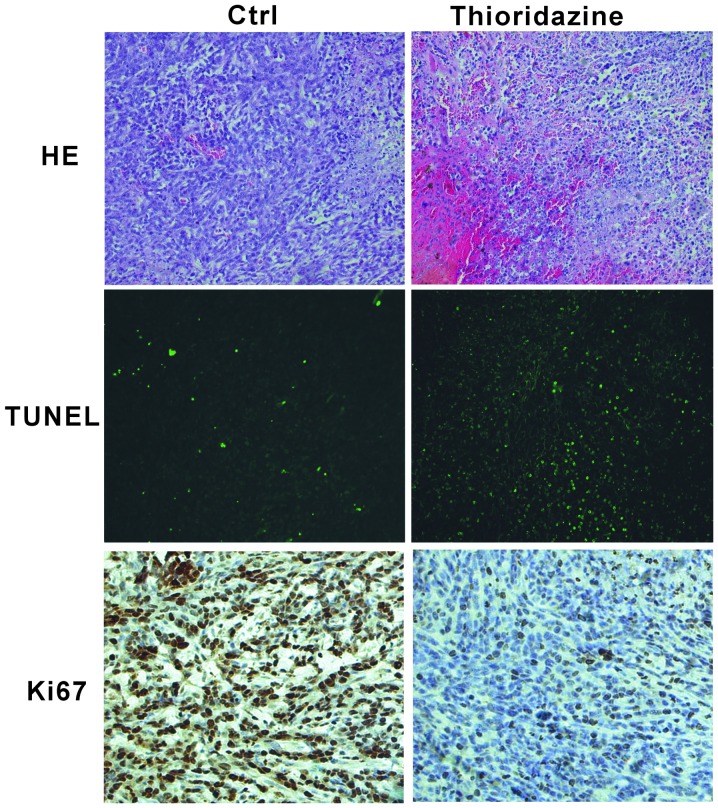
Thioridazine induces tumor necrosis and apoptosis and inhibits tumor cell proliferation. The upper panel shows HE-stained tumor sections, x200 magnification. Hemorrhagic necrosis was increased following treatment with thioridazine (right) compared with the control (left). The middle panel shows apoptosis detected using TUNEL staining. A higher frequency of apoptotic tumor cells was found in the tumors from the thioridazine-treated animals (right) compared with the controls (left). The lower panel shows tumor sections immunohistochemically stained for Ki67. A greater number of mitotic tumor cells was observed in the tumors from the control mice (left) compared with the tumors from the thioridazine-treated animals (right). HE, hematoxylin and eosin; TUNEL, terminal deoxynucleotidyl transferase dUTP nick end labeling; ctrl, control.

In order to investigate whether thioridazine inhibits tumor cell proliferation *in vivo*, histological assessment was performed using tumor samples from 4T1 tumors. The 4T1 tumors from the mice in the control group showed poor differentiation and limited necrosis (Fig. 3, upper panel). By contrast, treatment with thioridazine was found to induce marked hemorrhagic necrosis (Fig. 3, upper panel). The TUNEL assay revealed that apoptosis was higher in the thioridazine treatment group compared with that in the control group (Fig. 3, middle panel). Furthermore, immunohistochemistry showed a decrease in cell proliferation in the thioridazine therapy group, as indicated by a decrease in the cell cycle marker Ki67, with the tumors from the control mice exhibiting higher proliferation (Ki-67^+^) indexes (Fig. 3, lower panel).

### Thioridazine-induced suppression of the nuclear factor κ-light-chain-enhancer of activated B cells (NFκB) pathway in breast cancer cells

**Figure 4:**
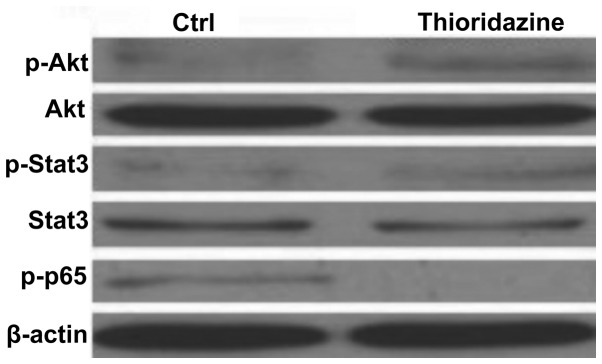
Thioridazine inhibits the NFκB pathway. 4T1 tumor cells were treated for 24 h with 20 µM thioridazine. Western blot analysis was performed using 4T1 tumor cell lysates and anti-p-Akt, -Akt, -p-STAT3, -STAT3 and p-NFκB p65 antibodies. β-actin was used as a loading control. Thioridazine did not affect the phosphorylation of Akt and STAT3, while it markedly inhibited phosphorylation of NFκB p65. STAT, signal transducer and activator of transcription; NFκB, nuclear factor κ-light-chain-enhancer of activated B cells; p-, phosphorylated; ctrl, control.

In order to determine the cell signaling pathways mediating the thioridazine-induced tumor inhibition in the 4T1 breast cancer cells, the levels of Akt, STAT3 and NFκB p65 were analyzed. As shown in Fig. 4, thioridazine was not observed to reduce the activity of Akt or STAT3 in 4T1 cells. However, thioridazine was found to significantly inhibit the phosphorylation of NFκB p65 (Fig. 4). These findings suggested that thioridazine inhibited cell proliferation and induced cell apoptosis through inhibiting the NFκB pathway.

### Thioridazine inhibits tumor angiogenesis

**Figure 5:**
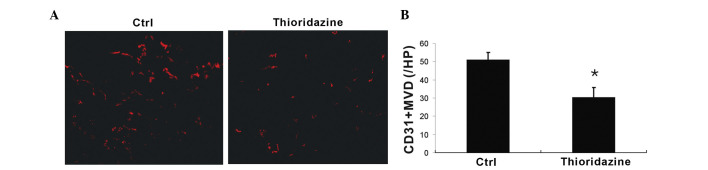
Thioridazine inhibits angiogenesis and reduces tumor vasculature. (A) Immunofluorecence staining with CD31 showed that more tumor vessels were present in the ctrl compared with the thioridazine-treated tumors. The vessels in thioridazine-treated tumors were smaller in diameter than those in the control tumors. (B) Quantification of CD31^+^ tumor vessels revealed that thioridazine significantly reduced tumor MVD. ^*^P<0.05 vs. ctrl. Ctrl, control; MVD, microvessel density.

In order to assess whether tumor blood vessel formation was inhibited by thioridazine treatment, CD31^+^ microvessels were analyzed using immunohistochemistry. Thioridazine was observed to markedly reduce vascularization compared with the control (Fig. 5A). Further more, the vessels in the thioridazine-treated tumors were found to be smaller in diameter than those in the control. Quantitative analysis of microvessel density revealed that thioridazine treatment had a significant inhibitory effect on tumor neovascularization (Fig. 5B). These findings suggested that thioridazine treatment inhibited tumor angiogenesis.

## Discussion

The present study investigated the anti-tumor efficacy of the DR inhibitor thioridazine in a mouse breast cancer model. Thioridazine was observed to significantly reduce tumor growth, inhibit proliferation and angiogenesis, as well as induce apoptosis. Furthermore, these thioridazine-induced effects were associated with inhibition of the canonical NFκB pathway.

Thioridazine has been extensively used to treat mental disorders. In the case of cancer, thioridazine has been used to treat tumor-associated depression (16) and sweating (17). In the present study, thioridazine was found to effectively suppress breast tumor growth in a preclinical model. Thioridazine has previously been identified to be an inhibitor of the phosphatidylinositol-3-kinase (PI3K)/Akt pathway in ovarian cancer cells (18). The PI3K pathway has an important role in tumorigenesis and therapeutic response (19). Almost every aspect of cell regulation, including apoptosis, proliferation, differentiation, oncogenic transformation, tumorigenesis and angiogenesis have been associated with PI3K activity (20). Thus, the present study investigated the effect of thioridazine on the inhibition of the PI3K/Akt pathway in breast cancer cells; however, thioridazine was not observed to affect Akt phosphorylation. Furthermore, thioridazine was not found to activate STAT3, another transcription factor associated with tumor proliferation and angiogenesis (21,22). The NFκB pathway has been identified to have a central role in apoptosis, proliferation and differentiation (23). The present study assessed the phosphorylation of the NFκB p65 subunit following treatment with thioridazine and found that thioridazine markedly reduced p65 phosphorylation. There are numerous NF-κB-dependent targets which are involved in different aspects of tumorigenesis, including c-Myc, cyclinD1, cyclinE and cyclin-dependent kinase 2 which are involved in tumor cell proliferation, X-linked inhibitor of apoptosis protein and survivin, which are involved in cell survival, matrix metalloproteinase 2/9 and intercellular adhesion molecule 1, which are involved in metastasis, interleukin (IL)-1, inducible nitric oxide synthase and cyclooxygenase 2, which are involved in inflammation, vimentin and twist, which are involved in EMT and vascular endothelial growth factor and IL-8, which are involved in angiogenesis (23). Several NFκB pathway inhibitors have been shown to be effective for cancer therapy (24).

Angiogenesis is essential for tumor growth and metastasis and targeting angiogenesis is a promising strategy for cancer therapy (25). The present study showed that thioridazine is an angiostatic agent which was found to effectively inhibit angiogenesis in a murine tumor model. There have been several studies on the anti-angiogenic effect of thioridazine, which have reported findings consistent with the results of the present study. *In vitro*, thioridazine has been observed to inhibit vascular endothelial growth factor (VEGF)-induced proliferation, invasion and endothelial cell tube formation of (26). In the present study, thioridazine was found to reduce microvessel density *in vivo*. Focal adhesion kinase (FAK) is upregulated in various types of cancer and has been reported to regulate tumor angiogenesis, which has been associated with endothelial cell survival, proliferation and migration (27). Thioridazine has been found to exert its anti-angiogenic effects through the inhibition of integrin αV-mediated VEGF expression in tumor cells and the inhibition of endothelial cell migration by suppressing the phosphorylation of Src/FAK (26). Furthermore, the angiostatic properties of thioridazine may be partially mediated through the suppression of CSCs. DRs are expressed on the CSCs (14), which secrete VEGF and have high angiogenic activity (28).

In conclusion, the present study provided evidence that the DR antagonist thioridazine is an effective drug against breast cancer. The potential for this drug to be used in cancer therapy requires further investigation in clinical settings.
